# Huge Arteriovenous Malformation of Upper Lip- A Case Report 

**DOI:** 10.22038/ijorl.2019.36848.2207

**Published:** 2020-01

**Authors:** Farzaneh Shobeirian, Morteza Sanei Taheri, Ruhollah Yeganeh, Hamidreza Haghighatkhah

**Affiliations:** 1 *Department of Radiology, Shohada-e-Tajrish Hospital, Shahid Beheshti University of Medical Sciences, Tehran, Iran.*; 2 *Department of Surgery, Loghman Hospital, Shahid Beheshti University of Medical Sciences, Tehran, Iran.*

**Keywords:** AVM, Digital subtraction angiography, Embolization, Lip AVM, Vascular embolization

## Abstract

**Introduction:**

Arteriovenous malformations (AVMs) are uncommon vascular lesions that can arise in any part of the body.

**Case Report:**

In this case study, we presented a huge AVM of the upper lip in a 70-year-old man that he noticed since 5years ago with slow growth in this period. Computed tomography angiography revealed a large AVM with feeders from the right facial artery and its branch superior labial artery. Right facial artery showed increased diameter and tortuous changes. Selective catheterization of right carotid was performed followed by super selective catheterization of the right facial artery. Then, the embolization of the tumor blush was conducted. Surgical removal of the tumor was carried out10days after the embolization.

**Conclusion:**

The AVM treatment is challenging, and there is a high chance of recurrence and progression. Every case should have an individualized approach that needs an accurate diagnosis and a multidisciplinary team.

## Introduction

According to the Mullikin and Glowacki classification, vascular lesions are divided into hemangiomas and vascular malformations. Vascular malformations consist of low-flow and high-flow lesions. Arteriovenous malformations (AVMs) are categorized as high-flow vascular lesions. The AVMs are uncommon vascular lesions that can be observed in every part of the body (1).

The AVMs can be life-threatening due to potential massive hemorrhage and/or cardiovascular instability (2,3). Facial AVMs are among abnormal fistulous connections between feeding arteries and draining veins. The draining veins are dilated and tortuous and may have variceal changes (3). Almost all patients with AVMs are children or adolescents (4). In this article, we reported a huge life-threatening AVM of the upper lip in a 70-year-old man and its treatment.

## Case Report

**Fig 1:**
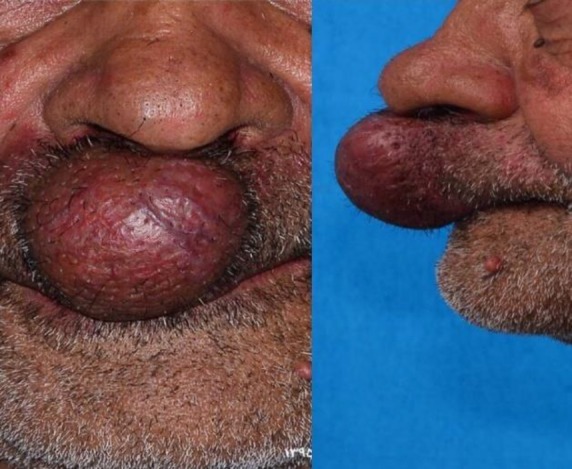
Reddish and violet mass-like facial lesion

A 70-year-old man presented to our general surgery clinic with a complaint of a slow-growing reddish and violet mass-like facial lesion in the upper lip (Fig.1). For the first time, he noticed the lesion 5 years ago. Recently, the lesion started to enlarge, and the patient felt pain in his upper lip and cheek area. There was no history of trauma in the area. He also denied any previous medical conditions. On physical examination, a purple mass approximately with a size of 6×6 cm was noticed on the upper lip extending to the nasolabial junction. Presence of bruit and slightly warm skin over the lesion suggested a presumptive diagnosis of a vascular lesion.

**Fig 2:**
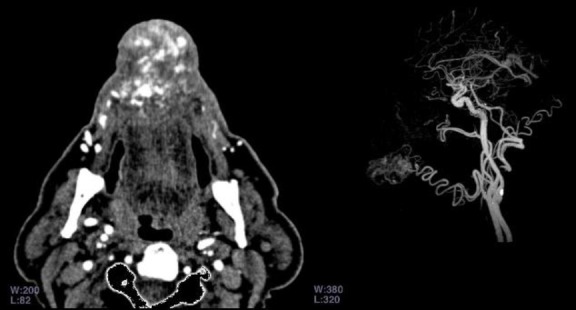
Arteriovenous malformation with feeders from the right facial artery and its superior labial branch artery

Ultrasound examination showed a high vascular mass with low-resistance spectrum characteristic of an AVM. Color Doppler examination also revealed large draining veins. Computed-Tomography (CT) showed a large soft tissue density lesion with the attenuation values of 40-50 HU. The CT angiography (CTA) revealed a large AVM with feeders from the right facial artery and its superior labial branch artery. The right facial artery showed increased diameter and tortuous changes (Fig.2).

**Fig 3:**
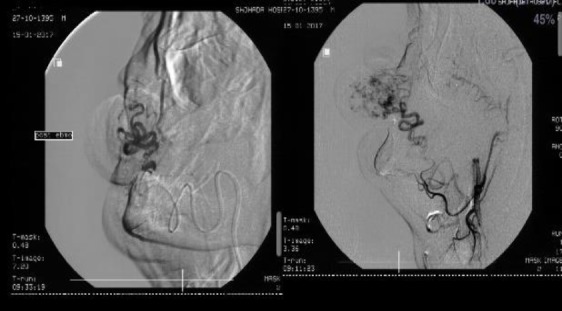
Super selective catheterization of the right facial artery

Vascular embolization and then surgical removal of the mass were planned considering the aforementioned clinical and radiological findings. Vascular access was obtained through the right common femoral artery. Selective catheterization of the right carotid was performed by multi-purpose catheter 5-Fr and a hydrophilic guidewire 6-Fr. the angiogram with nonionic contrast showed a large AVM with feeders from the right facial artery and its superior labial branch artery. Venous drainage was into the jugular vein. Afterward, the super selective catheterization of the right facial artery was performed proximally to the branch supplying the lesion. Embolization of tumor blush with embosphere microspheres of 300-500 µm was performed (Fig.3.).

**Fig 4:**
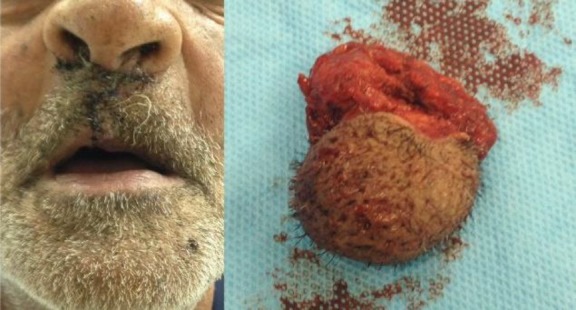
After arteriovenous malformation excision

Control angiography demonstrated no flow in the AVM. In addition, the procedure had no complication. The surgical intervention was performed 10days after the embolization, and a well-demarcated AVM lesion was removed without significant hemorrhage. Preoperative embolization made the AVM demarcated and firm in palpation. Regarding lip contour, lip closure was carefully conducted (Fig.4).There was no complication in the clinical follow-up. The AVM did not recur after 6months of follow-up.

## Discussion

There are two types of vascular anomalies, including vascular malformations and hemangiomas. Among all vascular anomalies, AVMs are considered to be the most uncertain and maybe the most dangerous ones most of which involve the head and neck (5,6). The AVMs of the head and neck are rare with no clear incidence. Vascular malformations are congenital lesions. Most AVMs become detectable until adolescence and visible due to hormones, infections, and traumas (6,7).The AVMs have an age range of 3 months to74 years. Acquired AVMs that present after trauma in contrast to congenital AVMs typically demonstrate a single arterial enlisting, which causetheireasier treatment (8).

The AVMs consist of a central nidus with abnormal shunts between arterial and venous systems that result in the dilation of adjacent arteries and veins. The AVMs have no proliferative cellular activity (3). Clinically, AVMs usually presents with warm firm compressible pulsatile painless slow-growing mass with bruits and trills (1,3,7). Ultrasound and Doppler ultrasound are used as radiologic examinations that can show vessels and extensions. Doppler ultrasound can provide the measurement of blood flow velocity and vessel resistance. The CT scan is very useful and usually shows soft tissue mass with enlarged adjacent arteries and veins (1). The CTA can help to confirm nidus and collateral circulation.

Magnetic resonance imaging is used to estimate extension and invasion to surrounding soft tissue based on contrast-enhanced T1-weighted and T2-weighted images (9). Magnetic resonance angiography provides pre-embolization planning with detecting the origin of anomalous branches (1). However, catheter angiography is the gold standard radiologic test. Accurate diagnosis is very important to develop an appropriate surgical plan. As the spontaneous regression of sporadic AVMs is rare, an effective therapeutic approach is important. Treatment of AVMs is controversial. Over the past decade, different approaches, including surgical excision, endovascular embolization, laser therapy, or a combination therapy have been used for the management of head and neck AVMs (10). The best success rates in AVM treatment have been reported with embolization followed by excisional surgery. The embolizingagents used are Onyx, Gel foam, coils, Glue, Embosphere, and polyvinyl alcohol (11). 

Some procedures, such as the proximal ligation of feeding arteries to AVM or curettage and partial resection, cause the recurrence of the lesion (6).Combination therapy has the highest success rate and is considered a gold standard therapy (12). Main goal of the treatment should be to eradicate the nidus and proximal of venous outflow. The choice is preoperative super selective arterial catheterization and embolization followed by surgery as soon as possible, ideally within 72 h (1,3,7,8).

## Conclusion

The AVM treatment is challenging with a high chance of recurrence and progression. Every case should have an individualized approach that requires an accurate diagnosis and a multidisciplinary team. Upper lip malformations, as well as other parts of the face, can be treated with the combination therapy of preoperative embolization and complete resection.
